# Cinnamaldehyde and Curcumin Prime Akt2 for Insulin-Stimulated Activation

**DOI:** 10.3390/nu14163301

**Published:** 2022-08-12

**Authors:** Yasuyo Urasaki, Thuc T. Le

**Affiliations:** College of Pharmacy, Roseman University of Health Sciences, 10530 Discovery Drive, Las Vegas, NV 89135, USA

**Keywords:** capillary electrophoresis, cinnamaldehyde, curcumin, diabetes, insulin, isoelectric focusing, protein phosphatase 2, post-translational modification, serine/threonine protein kinase, tyrosine phosphatase

## Abstract

In this study, the effects of cinnamaldehyde and curcumin on Akt2, a serine/threonine protein kinase central to the insulin signaling pathway, were examined in preadipocytes. Cinnamaldehyde or curcumin treatment increased Akt2 phosphorylation at multiple sites including T450 and Y475, but had no effect on Akt2 phosphorylation at S474, which is critical for Akt2 activation. Surprisingly, insulin treatment with cinnamaldehyde or curcumin increased p-Akt2 (S474) by 3.5-fold versus insulin treatment alone. Furthermore, combined cinnamaldehyde, curcumin, and insulin treatment increased p-Akt2 (S474) by 7-fold versus insulin treatment alone. Interestingly, cinnamaldehyde and curcumin inhibited both serine/threonine phosphatase 2A (PP2A) and protein tyrosine phosphatase 1B (PTP1B). Akt2 activation is a multistep process that requires phosphorylation at T450 for proper folding and maturation, and phosphorylation of both Y475 and S474 for stabilization of the catalytic domain. It is plausible that by inhibiting PP2A and PTP1B, cinnamaldehyde and curcumin increase phosphorylation at T450 and Y475, and prime Akt2 for insulin-stimulated phosphorylation at S474. Notably, the combination of a PP2A inhibitor, okadaic acid, and a PTP1B inhibitor increased p-Akt2 (S474), even in the absence of insulin. Future combinations of PP2A and PTP1B inhibitors provide a rational platform to engineer new therapeutics for insulin resistance syndrome.

## 1. Introduction

Phytonutrients, or natural compounds found in plants, are known to have anti-diabetic effects [1]. For example, cinnamaldehyde from the bark of cinnamon trees and curcumin from the rhizomes of turmeric plants are insulin-sensitizing phytonutrients [2,3]. Both cinnamaldehyde and curcumin have beneficial effects for the management of hyperglycemic condition in patients with type 2 diabetes [4,5]. However, vaguely understood mechanisms of action hinder their therapeutic potential.

This study examines the effects of cinnamaldehyde and curcumin on Akt serine/threonine kinase 2, or Akt2. The Akt kinase family comprises three highly homologous isoforms of Akt1, Akt2, and Akt3, which exhibit distinctive functional specificity and tissue distribution [6,7]. Akt1 is ubiquitously expressed in all tissue types and regulates cell growth and survival [8]. Akt2 is primarily expressed in insulin-responsive tissues such as skeletal muscle and adipose tissues and regulates glucose metabolism [9]. Akt3 is predominantly expressed in nervous tissues and regulates neuronal development [10]. Interestingly, a missense mutation in Akt2 is reported in a family with severe insulin resistance and diabetes mellitus [11]. Akt2 isoform-specific targeting is a viable therapeutic approach toward the management of glucose metabolism disorders [12].

In recent years, capillary isoelectric focusing (cIEF) immunoassay has provided a robust means to study Akt isoform-specific regulation [13,14,15]. cIEF separates proteins in individual capillaries by their isoelectric points (pI) [16]. Subsequent immunoassays using antibodies specific to an Akt isoform permit resolving its distribution as a function of pI values [17]. Change to the pI value of a protein indicates post-translational modifications (PTMs) [18]. For example, phosphorylation or acetylation of a protein causes its pI to shift toward lower values [19]. In contrast, glycosylation of a protein causes its pI to shift toward higher values [20]. cIEF immunoassays permit rapid assessment of the PTM profiles of Akt isoforms in response to extracellular stimulation [21]. Herein, cIEF immunoassays are deployed to measure PTMs of Akt2 following the treatment of primary human subcutaneous preadipocytes with insulin, cinnamaldehyde, or curcumin.

## 2. Materials and Methods

### 2.1. Primary Human Subcutaneous Preadipocytes

Primary human subcutaneous preadipocytes were obtained from Zen-Bio (Cat. No. SP-F-1, Lot #L031219A, Durham, NC, USA) and maintained at 37 °C and 5% CO_2_ in growth medium comprising Minimum Essential Medium α (cat. no. 12571063, Thermo Fisher Scientific, Waltham, MA, USA) supplemented with 10% fetal bovine serum, 100 units/mL penicillin, and 100 µg/mL streptomycin. Primary human subcutaneous preadipocytes were isolated from the thigh white adipose tissues of a healthy Caucasian female who was 43 years old and had a BMI < 24.9. The donor was undergoing elective surgery and signed an Institutional Review Board validated donor consent form that specifically lists both the intended uses for non-clinical research and confirms the procedures for processing the samples are Standard Operating Procedure managed Good Laboratory Practices protocols in compliance with ethical regulations. All samples were collected and processed in the United States.

### 2.2. Treatment Conditions

Existing growth media of primary human subcutaneous preadipocytes were replaced with new growth media premixed with treatment compounds and incubated at 37 °C and 5% CO_2_ for 30 min prior to the collection of total cell extracts. The final concentrations of treatment compounds were as follows: insulin (100 nM, Humulin R, Eli Lilly, Indianapolis, IN, USA), cinnamaldehyde (40 µM, cat. no. W228613, Sigma Aldrich, St. Louis, MO, USA), curcumin (20 µM, cat. no. C7727, Sigma Aldrich, St. Louis, MO, USA), okadaic acid (5 nM, cat. no. ab120375, Cambridge, MA, USA), and PTP1Bi (4 µM, cat. no. 15782, Cayman Chem, Ann Arbor, MI, USA). The concentrations of treatment compounds were selected based on their ability to induce maximal effects on the PTM profile of Akt2, which were experimentally determined. Maximal effect on the PTM profile of Akt2 was observed at 30 min post-treatment for all treatment compounds.

### 2.3. Preparation of Total Cell Extracts

Approximately one million cells were incubated on ice for 10 min with 60 µL of lysis buffer (cat. no. 040-764, ProteinSimple, Santa Clara, CA, USA), sonicated 4 times for 5 s each, mixed by rotation for 2 h at 4 °C, and centrifuged at 12,000 rpm in an Eppendorf 5430R microfuge for 20 min at 4 °C. The supernatant was collected as the cell lysate. The total protein concentration in the cell lysate was determined with a Bradford protein assay and adjusted to a final concentration of 0.3 µg/µL with separation gradients (cat. no. Premix G2, pH 5–8, ProteinSimple, Santa Clara, CA, USA) for charge-based cIEF immunoassays.

### 2.4. Capillary Isoelectric Focusing Immunoassays

Cell lysates in separation gradients were loaded into 384-well assay plates (cat. no. 040-663, ProteinSimple, Santa Clara, CA, USA), which were preloaded with primary and secondary antibodies and chemiluminescent substrates. Charge-based protein separation and detection in individual capillaries were performed using the default protocols of the NanoPro 1000 system (ProteinSimple, Santa Clara, CA, USA). HSP70 was used as the loading control. All cIEF immunoassays were performed in triplicate for each protein, and triplicate experiments were performed for each treatment condition, producing nine repeated measurements per protein analyte.

### 2.5. Capillary Western Immunoassays

The total protein concentration in the cell lysate was determined with a Bradford protein assay and adjusted to a final concentration of 0.4 µg/µL with denaturing buffers (cat. no. PS-ST01EZ or PS-ST03EZ, ProteinSimple, Santa Clara, CA, USA) for size-based Western immunoassays. Cell lysates in denaturing buffers were denatured at 95 °C for 5 min, and then transferred to assay plates (cat. no. SM-W004 or SM-W008, ProteinSimple, Santa Clara, CA, USA) preloaded with blocking reagents, wash buffer, primary and secondary antibodies, and chemiluminescent substrates. Sized-based protein separation and detection in capillaries were performed using the default protocols of the Jess system (ProteinSimple, Santa Clara, CA, USA). β-actin was used as a loading control. All capillary Western immunoassays were performed in triplicate for each protein, and duplicate experiments were performed for each treatment condition, producing six repeated measurements per protein analyte.

### 2.6. Antibodies

Primary antibodies used for this study were: pan-Akt (cat. no. 8312, Santa Cruz Biotech, Dallas, TX, USA), Akt 1 (cat. no. 2938, cat. no. 2938, Cell Signaling Technology (CST), Danvers, MA, USA), Akt2 (cat. no. cs3063, CST), Akt3 (cat. no. 8018, CST), p-Akt (S473) (cat. no. 9271, CST), p-Akt1 (S473) (cat. no. 9018, CST), p-Akt2 (S474) (cat. no. cs8599, CST), p-Akt (S124) (cat. no. PA5-38251, Thermo Fisher Scientific, Waltham, MA, USA), p-Akt (S246) (cat. no. PA5-38352, Thermo Fisher Scientific, Waltham, MA, USA), p-Akt (T308) (cat. no. cs2965, CST), p-Akt (T450) (cat. no. cs9267, CST), p-Akt (Y475) (p-Akt1 (Y474) or p-Akt2 (Y475), cat. no. PA5-38351, Thermo Fisher Scientific, Waltham, MA, USA), HSP70 (cat. no. cs4872, CST), and β-actin (cat. no. MAB8929, R&D Systems, Minneapolis, MN, USA). Horse radish peroxidase secondary antibodies were used for this study (cat. no. 040-656, 042-205, 042-206, 043-819, and 043-821, ProteinSimple, Santa Clara, CA, USA).

### 2.7. Data Analysis

Protein abundance was quantified using the Compass software from ProteinSimple (Santa Clara, CA, USA) and normalized with that of HSP70 for cIEF immunoassays or β-actin for capillary Western assays. HSP70 and β-actin served as loading controls.

### 2.8. Phosphatase Assays

Ser/Thr phosphatase assay kit (cat. no. 17-127, Millipore, Temecula, CA, USA) was used to detect PP2A activity by dephosphorylation of the phosphopeptide R-K-pT-I-R-R using malachite green detection. Likewise, a protein tyrosine phosphatase assay kit (cat. no. 17-125, Millipore, Burlington, MA, USA) was used to detect PTP1B activity by dephosphorylation of the phosphopeptide R-R-L-I-E-D-A-E-pY-A-A-R-G using malachite green detection. Experimental procedures were performed according to the manufacturer’s protocols. Recombinant PP2A (cat. no. 10011237, Cayman Chem, Ann Arbor, MI, USA) and PTP1B (cat. no. 10010896, Cayman Chem, Ann Arbor, MI, USA) were used. Dephosphorylation reactions were performed at room temperature for 30 min in the presence of serially diluted okadaic acid, PTP1Bi, cinnamaldehyde, or curcumin. The reactions were terminated by the addition of 100 µM malachite green solution and allowed color development to proceed for 15 min at room temperature. Phosphatase activities were measured using absorbance at 650 nm in a microtiter plater reader (Synergy 2, BioTek, Winooski, VT, USA).

### 2.9. Statistical Analysis

Quantitative data were presented as the mean values ± standard deviations across nine repeated measurements. Statistical significance was calculated with Student’s *t*-test and thresholded at *p* ≤ 0.01 versus the control.

## 3. Results

### 3.1. Composition of Akt Isoforms in Primary Human Subcutaneous Preadipocytes

**Figure 1:**
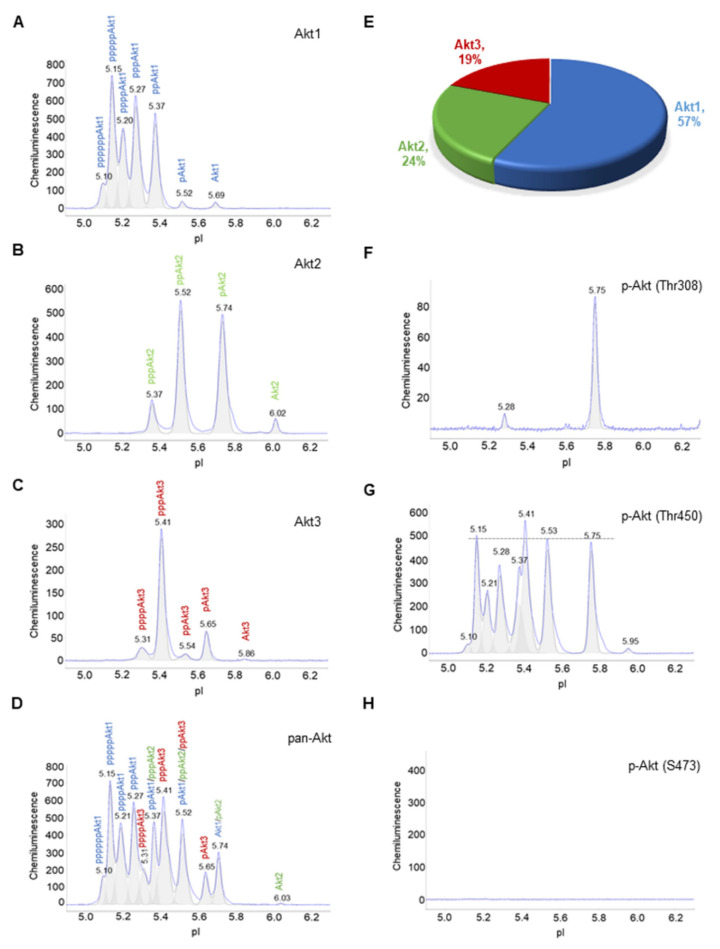
Composition of Akt isoforms in primary human subcutaneous preadipocytes. (**A**–**D**) Electropherograms of capillary isoelectric focusing (cIEF) immunoassays of (**A**) Akt1, (**B**) Akt2, (**C**) Akt3, and (**D**) pan-Akt or all Akt isoforms. The letter p represents phosphorylation of Akt isoforms. Peaks on electropherograms are color-coded with Akt1 (blue), Akt2 (green), and Akt3 (red). (**E**) Percentage of Akt1 (blue), Akt2 (green), and Akt3 (red) as a function of all Akt isoforms. (**F**–**H**) Electropherograms of cIEF immunoassays of (**F**) pAkt (Thr308), (**G**) pAkt (Thr450), and (**H**) pAkt (S473). The dashed line highlights the distribution of pAkt (Thr450) as a function of pI values.

Akt isoforms and selective phosphorylation sites were examined using cIEF immunoassays in primary human subcutaneous preadipocytes. Specifically, primary antibodies that recognize Akt1, Akt2, Akt3, and pan-Akt or all Akt isoforms were used (Figure 1A–D). The distribution of phosphoisoforms as functions of pI for each Akt isoform was clearly observed on cIEF electropherograms (Figure 1A–C). The composition of each Akt isoform as a function of all Akt isoforms was resolvable on the pan-Akt electropherogram (Figure 1D). On average, Akt1, Akt2, and Akt3 isoforms were 57%, 24%, and 19% of all Akt isoforms, respectively (Figure 1E). In addition, primary antibodies that recognize phosphoisoforms of pan-Akt were used for cIEF immunoassays. Phosphoisoform p-Akt (T308) was detectable mainly at pI 5.74, which coincided with a major peak on the Akt2 electropherogram (Figure 1F). Phosphoisoform p-Akt (T450) was highly expressed and evenly distributed across a broad range of pI values, which coincided with most peaks on all Akt isoforms (Figure 1G). In contrast, phosphoisoform p-Akt (S473) was undetectable (Figure 1H).

### 3.2. Insulin Induces Changes to the Electropherograms of Akt2 and Akt3

**Figure 2:**
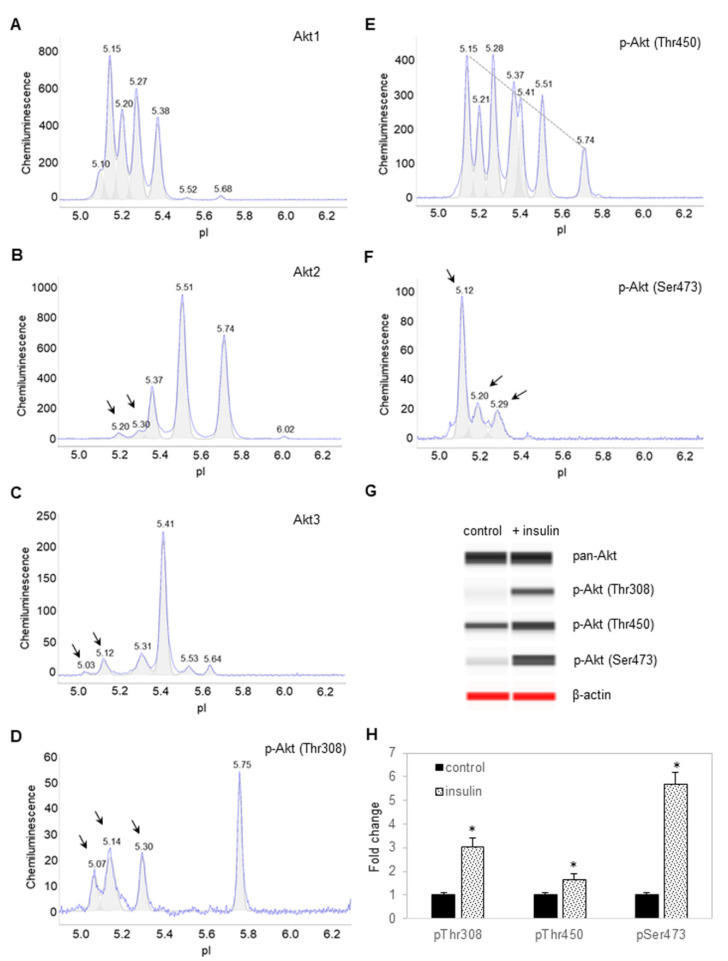
Insulin induces changes to the electropherograms of Akt2 and Akt3. Electropherograms of cIEF immunoassays of (**A**) Akt1, (**B**) Akt2, (**C**) Akt3, (**D**) p-Akt (Thr308), (**E**) p-Akt (Thr450), and (**F**) p-Akt (Ser473) following treatment with insulin for 30 min. Arrows point to new peaks that appeared after treatment with insulin. The dashed line highlights the distribution of p-Akt (Thr450) as a function of pI values. (**G**) Capillary Western (CW) immunoassays of p-Akt (Thr308), p-Akt (Thr450), and p-Akt (Ser473) before and after treatment with insulin. Pan-Akt and β-actin served as the loading controls. (**H**) Relative expression level of p-Akt (Thr308), p-Akt (Thr450), and p-Akt (Ser473) before (solid black) and after (textured) treatment with insulin. Error bars indicate standard deviations across six repeated measurements using CW immunoassays per experimental condition. Asterisks indicate a statistical significance of *p* ≤ 0.01 versus untreated control.

Following the treatment of preadipocytes for 30 min with insulin, Akt isoforms and selective phosphorylation sites were again examined using cIEF immunoassays. Surprisingly, insulin treatment had no observable effect on the electropherograms of Akt1 isoform (Figure 2A). In contrast, insulin treatment induced the appearance of new Akt2 and Akt3 isoforms at low pI values (Figure 2B,C). Insulin treatment induced the appearance of new peaks at low pI values for p-Akt (T308) (Figure 2D). Insulin treatment also changed the distribution of p-Akt (T450) isoforms, where the relative abundance of peaks at low pI values were higher versus those at higher pI values (Figure 2E). Expectedly, insulin treatment induced the appearance of new peaks at low pI values for p-Akt (S473) (Figure 2F). Capillary Western immunoassays revealed that insulin treatment increased the abundance of p-Akt (T308), p-Akt (T450), and p-Akt (S473) (Figure 2G,H). Phosphorylation of Akt isoforms generally induced new peaks at low pI values. On the one hand, the abundance of peaks at low pI values on the electropherogram of Akt1 most likely obscured the effect of insulin-stimulated phosphorylation. On the other hand, the appearance of new peaks at low pI values following insulin treatment was readily detectable on the electropherograms of Akt2 and Akt3 (Figure 2A,B).

### 3.3. Cinnamaldehyde and Curcumin Induce Posttranslational Modification of Akt2

**Figure 3:**
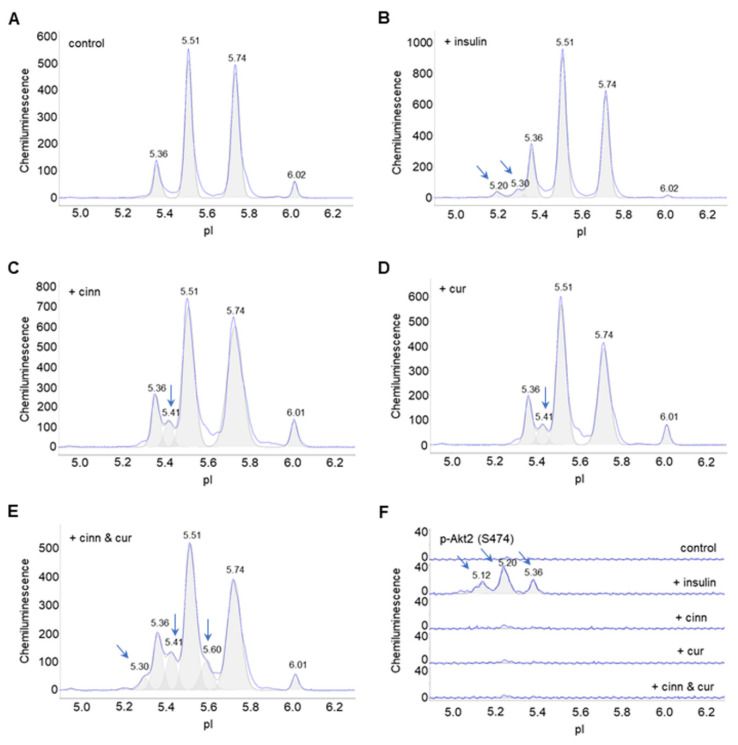
Cinnamaldehyde and curcumin induce changes to Akt2 posttranslational modification. (**A**–**E**) Distribution of Akt2 as a function of isoelectric points in (**A**) control untreated preadipocytes, or (**B**–**E**) preadipocytes treated with (**B**) insulin, (**C**) cinnamaldehyde, (**D**) curcumin, or (**E**) combined cinnamaldehyde and curcumin. (**F**) Distribution of p-Akt2 (S474) as a function of isoelectric points in untreated control preadipocytes (top electropherogram), or preadipocytes treated with insulin (second electropherogram), cinnamaldehyde (third electropherogram), curcumin (fourth electropherogram), and combined cinnamaldehyde and curcumin (bottom electropherogram). Arrows point to the appearance of new peaks following treatment versus untreated control.

Next, the high-throughput capability of cIEF immunoassays was deployed to screen for the effects of hundreds of phytonutrients on the PTM of Akt1, Akt2, and Akt3 in preadipocytes. Cinnamaldehyde and curcumin were identified as two phytonutrients that induced changes to the electropherogram of Akt2. Specifically, cinnamaldehyde and curcumin induced the appearance of a new peak at pI 5.41 on Akt2 electropherograms that was distinct from the peaks induced with insulin treatment at pI 5.20 and 5.30 (Figure 3A–D). Combined cinnamaldehyde and curcumin treatment induced the appearance of multiple new Akt2 isoforms at pI 5.60, 5.41, and 5.30 (Figure 3E). On the p-Akt2 (S474) electropherogram that specifically detects phosphorylated S474 residue of Akt2 isoform, only insulin treatment induced the appearance of p-Akt2 (S474), which is critical for Akt2 activation (Figure 3F). Neither untreated control preadipocytes or preadipocytes treated with cinnamaldehyde or curcumin had a detectable level of p-Akt2 (S474). Surprisingly, neither cinnamaldehyde or curcumin had any observable effect on the electropherograms of Akt1 or Akt3 (Figure S1A,B).

### 3.4. Cinnamaldehyde and Curcumin Enhance Insulin-Stimulated Activation of Akt2

**Figure 4:**
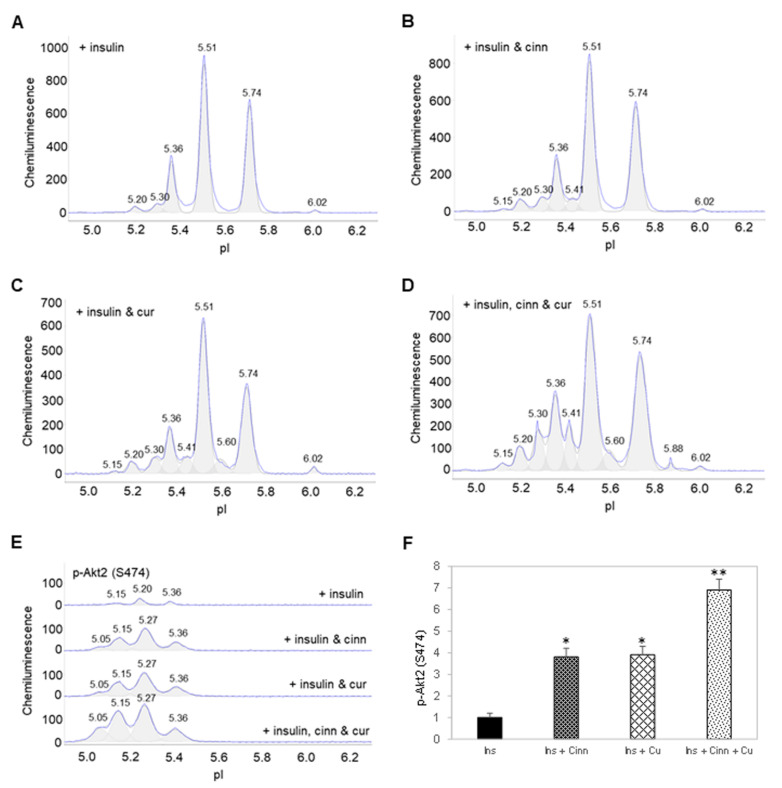
Cinnamaldehyde and curcumin enhance insulin-stimulated activation of Akt2. (**A**–**D**) Distribution of Akt2 as a function of isoelectric points in preadipocytes treated with (**A**) insulin alone, (**B**) insulin and cinnamaldehyde, (**C**) insulin and curcumin, or (**D**) insulin, cinnamaldehyde and curcumin. (**E**) Distribution of p-Akt2 (S474) as a function of isoelectric points in preadipocytes treated with insulin alone (top electropherogram), insulin and cinnamaldehyde (second electropherogram), insulin and curcumin (third electropherogram), or insulin, cinnamaldehyde, and curcumin (bottom electropherogram). (**F**) Relative abundance of p-Akt2 (S474) as a function of treatment condition. Error bars are standard deviations across nine repeated measurements. Single asterisk (*) indicates *p*-value ≤ 0.01 versus treatment with insulin alone. Double asterisk (**) indicates *p*-value ≤ 0.01 versus treatment with insulin and cinnamaldehyde or insulin and curcumin.

Interestingly, insulin treatment together with either cinnamaldehyde or curcumin induced the appearance of a new Akt2 isoform at pI 5.15 and increased the abundance of Akt2 isoforms at pI 5.20 and 5.30 (Figure 4A–C). Insulin treatment together with both cinnamaldehyde and curcumin further increased the abundance of Akt2 isoforms from pI 5.15 to 5.41 (Figure 4D). Most significantly, combined insulin and cinnamaldehyde treatment or insulin and curcumin increased p-Akt2 (S474) by approximately 3.5-fold compared to insulin treatment alone (Figure 4E,F). Additionally, combined insulin, cinnamaldehyde, and curcumin treatment increased p-Akt2 (S474) by nearly 7-fold compared to insulin treatment alone. Clearly, cinnamaldehyde and curcumin enhanced insulin-stimulated activation of Akt2.

### 3.5. Cinnamaldehyde and Curcumin Increase Pan-Akt Phosphorylation

**Figure 5:**
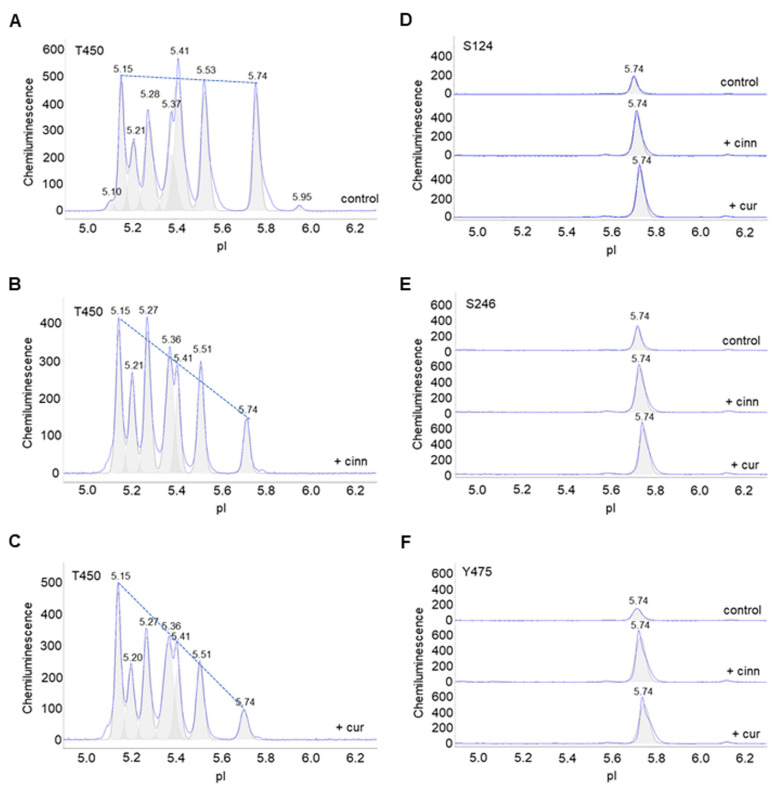
Cinnamaldehyde and curcumin increase pan-Akt phosphorylation. (**A**–**C**) Distribution of p-Akt (T450) as a function of isoelectric points in (**A**) untreated control preadipocytes or (**B**,**C**) preadipocytes treated with (**B**) cinnamaldehyde or (**C**) curcumin. The dashed line highlights the distribution trend. (**D**–**F**) Distribution of (**D**) p-Akt (S124), (**E**) p-Akt (S246), and (**F**) p-Akt (Y475) in untreated control preadipocytes (top electropherogram), or preadipocytes treated with cinnamaldehyde (middle electropherogram) or curcumin (bottom electropherogram).

Furthermore, the effects of cinnamaldehyde and curcumin on other phosphorylation sites were examined. Due to the unavailability of Akt2-specific antibodies, antibodies that recognize p-Akt (S124), p-Akt (S246), p-Akt (T308), p-Akt (T450), and p-Akt (Y475) of all Akt isoforms were used. p-Akt (T450) was highly expressed and evenly distributed across a broad range of pI values in control untreated preadipocytes. Cinnamaldehyde or curcumin treatment changed the distribution of p-Akt (T450) isoforms, where the relative abundance of peaks at low pI values were higher versus those at higher pI values (Figure 5A–C). This observation indicates increased phosphorylation at the T450 residue. Similarly, cinnamaldehyde or curcumin treatment increased the abundance of p-Akt (S124), p-Akt (S246), and p-Akt (Y475) at pI 5.74 compared to untreated control (Figure 5D–F). Interestingly, cinnamaldehyde or curcumin treatment had no observable effect on p-Akt (T308), which is also associated with the activation of Akt (Figure S2). Collectively, these data concurred that cinnamaldehyde and curcumin increased Akt phosphorylation, but not its activation (Figures S3 and S4).

### 3.6. Cinnamaldehyde and Curcumin Inhibit Both PP2A and PTP1B

**Figure 6:**
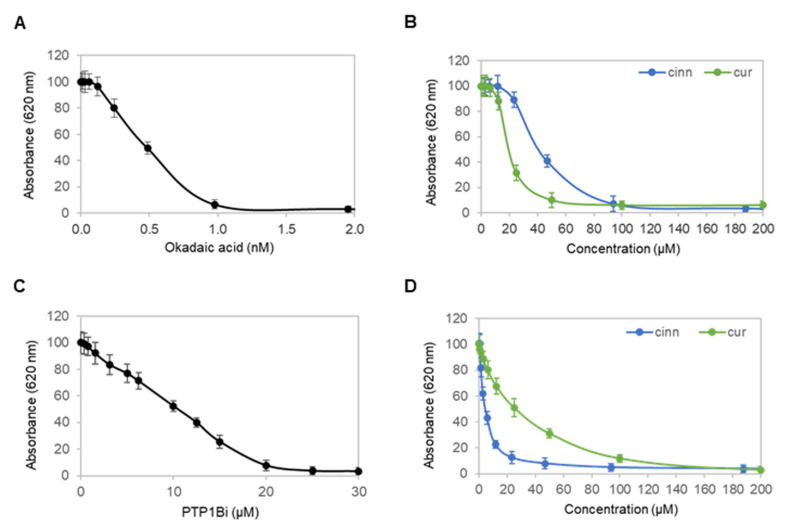
Cinnamaldehyde and curcumin inhibit both PP2A and PTP1B. (**A**,**B**) Activity of a recombinant protein serine/threonine phosphatase PP2A as a function of titrating concentrations of (**A**) okadaic acid, or (**B**) cinnamaldehyde (blue) and curcumin (green). (**C**,**D**) Activity of a recombinant protein tyrosine phosphatase PTP1B as a function of titrating concentrations of (**C**) PTP1Bi, or (**D**) cinnamaldehyde (blue) and curcumin (green). Error bars are standard deviations across nine repeated measurements.

Cinnamaldehyde and curcumin are known inhibitors of protein serine/threonine phosphatase 2A (PP2A) and protein tyrosine phosphatase 1B (PTP1B) [22,23]. Next, enzymatic activities of recombinant PP2A and PTP1B proteins were measured in the presence of cinnamaldehyde and curcumin and compared to okadaic acid, a known PP2A inhibitor [24], and a PTP1B inhibitor (PTP1Bi) [25]. Okadaic acid inhibited PP2A with a half-maximal inhibitory concentration (IC_50_) of approximately 0.5 nM (Figure 6A). By comparison, cinnamaldehyde and curcumin inhibited PP2A with IC_50_ values of approximately 40 and 20 µM, respectively (Figure 6B). Expectedly, PTP1Bi inhibited PTP1B at an IC_50_ of approximately 10 µM (Figure 6C). By comparison, cinnamaldehyde and curcumin inhibited PTP1B at an IC_50_ of approximately 4 and 25 µM, respectively (Figure 6D). Taken together, cinnamaldehyde and curcumin inhibited both PP2A and PTP1B.

### 3.7. Dual Inhibition of PP2A and PTP1B Activates Akt2

**Figure 7:**
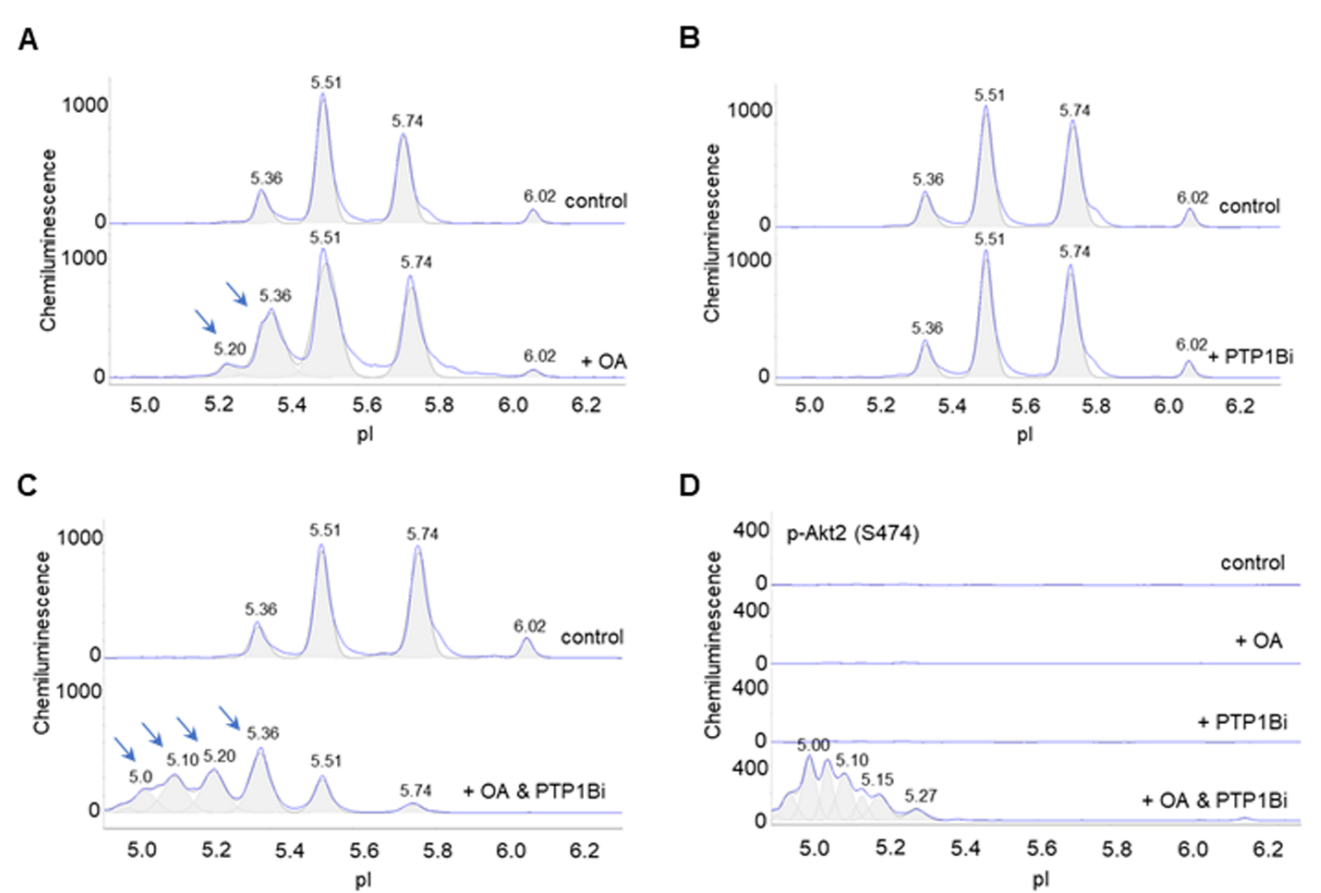
Dual inhibition of PP2A and PTP1B activates Akt2. (**A**–**C**) Distribution of Akt2 as a function of isoelectric points in control untreated preadipocytes (top electropherograms) or in preadipocytes treated with (**A**) okadaic acid, (**B**) PTP1Bi, or (**C**) combined okadaic acid and PTP1B (bottom electropherograms). Arrows point to new peaks or peaks that experience elevated abundance in treated preadipocytes versus untreated control preadipocytes. (**D**) Distribution of p-Akt2 (S474) as a function of isoelectric point in control untreated preadipocytes (top electropherogram), or preadipocytes treated with okadaic acid (second electropherogram), PTP1Bi (third electropherogram), or combined okadaic acid and PTP1Bi (bottom electropherogram).

Lastly, the effects of okadaic acid and PTP1Bi on Akt2 were examined in primary human subcutaneous preadipocytes. On the one hand, okadaic acid treatment induced the appearance of a new Akt2 isoform at pI 5.20 and increased the abundance of an existing Akt2 isoform at pI 5.36 versus the untreated control (Figure 7A). On the other hand, PTP1Bi treatment had no observable effect on the cIEF electropherogram of Akt2 versus the untreated control (Figure 7B). Surprisingly, combined okadaic acid and PTP1Bi treatment strongly induced the appearance of multiple Akt2 isoforms at low pI values from 4.90 to 5.20 (Figure 7C). Neither okadaic acid nor PTP1Bi treatment individually was capable of inducing p-Akt2 (S474) expression. In contrast, combined okadaic acid and PTP1Bi treatment strongly induced the expression of p-Akt2 (S474) (Figure 7D). Notably, the combined okadaic acid and PTP1B treatment activated Akt2 in the absence of insulin.

## 4. Discussion

The structure and activation mechanism of all three Akt isoforms are highly conserved [26]. All Akt kinases consist of three conserved domains, an *N*-terminal pleckstrin homology (PH) domain, a central kinase catalytic (CAT) domain, and a *C*-terminal extension (EXT) domain containing a regulatory motif. Amino acid sequence homology for the PH, CAT, and EXT domains among Akt isoforms are approximately 80%, 90%, and 70%, respectively [27]. Most significantly, amino acid residues whose phosphorylation are required for activation are conserved in all Akt isoforms [28]. Inferring from numerous studies on the activation mechanism of Akt1, Akt2 activation is expected to follow a similar multistep process [28], where constitutive phosphorylation of T450 and Y475 residues are prerequisites for subsequent activation by the phosphorylation of S474 [29,30,31,32]. Phosphorylation at the T450 residue controls Akt2 protein folding and maturation [33]. Furthermore, phosphorylation at both Y475 and S474 residues stabilizes the catalytic domain [31].

Cinnamaldehyde and curcumin preferentially target selective Akt isoforms. Cinnamaldehyde and curcumin promote insulin-stimulated activation of both Akt1 and Akt2. The abundance of peaks at low pI values on cIEF electropherograms of Akt1 hinders the detection of the effects of cinnamaldehyde, curcumin, or insulin on Akt1 phosphorylation. However, both cIEF immunoassays of p-Akt1 (S473) and standard Western immunoassays reveal that cinnamaldehyde and curcumin promote insulin-stimulated phosphorylation of Akt1 (Figure S5). Individually, cinnamaldehyde or curcumin treatment increases insulin-stimulated activation of Akt2 by approximately 3.5-fold versus insulin treatment alone. Together, cinnamaldehyde and curcumin treatment increases insulin-stimulated activation of Akt2 by nearly 7-fold versus insulin treatment alone. Surprisingly, cinnamaldehyde and curcumin have no observable effect on insulin-stimulated phosphorylation of Akt3 (Figure S6). In preadipocytes, Akt3 is a minor isoform, which constitutes approximately 19% of all Akt isoforms. The composition of Akt3 decreases to 7% of total Akt isoforms following the differentiation of preadipocytes into adipocytes [7,34]. Akt isoforms are present at distinct subcellular locations [35]. Akt1 is detectable at the plasma membrane, cytoplasm, and nucleus. Akt2 is localized to the mitochondrial membrane. Akt3 is localized to the nucleus and nuclear membrane. Distinctive spatial distribution of Akt isoforms could be a mechanism underlying their differential responses to cinnamaldehyde and curcumin, although further investigation is warranted.

Furthermore, cinnamaldehyde and curcumin have non-specific and multi-targeted effects. The effects of cinnamaldehyde and curcumin on Akt2 are not mediated by the insulin signaling pathway. The insulin signaling pathway is mediated by the PI3K/PDK1/Akt signaling axis. Inhibition of pI3K with an inhibitor LY294002 abrogates insulin-stimulated phosphorylation of Akt2 (Figure S7A). Interestingly, LY294002 treatment is unable to inhibit cinnamaldehyde or curcumin-induced phosphorylation of Akt2 (Figure S7B). Consistent with the literature on the positive regulatory effects of cinnamaldehyde or curcumin on the MAPK signaling pathway [36,37], increased ERK1/2 phosphorylation following the treatment with cinnamaldehyde, or curcumin, is also observed using cIEF immunoassays (Figure S8**).** Furthermore, both cinnamaldehyde and curcumin are reported to increase AMPK phosphorylation in several previous studies [38,39]. Given the structural dissimilarity between Akt2, ERK1/2, and AMPK, direct interaction between cinnamaldehyde or curcumin with these protein kinases is highly unlikely.

This study presents an alternative mechanism of action underlying the insulin-sensitizing effects of cinnamaldehyde and curcumin. As inhibitors of both PP2A and PTP1B, cinnamaldehyde and curcumin increase the abundance of phosphorylated T450 and Y475 residues and promote insulin-stimulated phosphorylation at the S474 residue of Akt2. Several molecular docking studies in recent years supported direct binding of cinnamaldehyde and curcumin to protein serine/threonine phosphatases and/or protein tyrosine phosphatases [40,41]. Both PP2A and PTP1B are negative regulators of the insulin signaling pathway [42,43], as well as other signaling pathways [44]. PP2A forms stable complexes with Akt [45,46,47,48], whereas PTP1B is a substrate of Akt [49]. Insulin resistance is a hallmark of type 2 diabetes mellitus [50]. Inhibition of PP2A or PTP1B is a viable approach toward improving insulin sensitivity for anti-diabetes therapy [42,43]. The utility of cinnamaldehyde and curcumin for diabetes prevention and management merits further investigation. Notably, the combination of okadaic acid and PTP1Bi strongly activates Akt2, even in the absence of insulin. Future combinations of PP2A and PTP1B inhibitors provide a rational platform to engineer new therapeutics for insulin resistance syndrome.

## 5. Patents

A U.S. patent application has been filed on behalf of the authors on a composition of phytonutrients for the management of diabetes that comprises cinnamaldehyde and curcumin (17/741,162).

## Data Availability

All data supporting the findings of this study are available from the corresponding author upon reasonable request.

## Supplementary Materials

The following supporting information can be downloaded at: https://www.mdpi.com/article/10.3390/nu14163301/s1, Figure S1. Cinnamaldehyde and curcumin have no observable effect on Akt1 and Akt3; Figure S2. Insulin induces the appearance of p-Akt (T308); Figure S3. Cinnamaldehyde and curcumin increase p-Akt (T450) isoform; Figure S4. Cinnamaldehyde and curcumin enhance insulin-stimulated phosphorylation of Akt2 (S474); Figure S5. Cinnamaldehyde and curcumin enhance insulin-stimulated activation of Akt1; Figure S6. Cinnamaldehyde and curcumin have no effect on insulin-stimulated phosphorylation of Akt3; Figure S7. The effects of cinnamaldehyde and curcumin are independent of the insulin signaling pathway; Figure S8. Cinnamaldehyde and curcumin promote ERK1/2 phosphorylation.

## References

[1] Martel J., Ojcius D.M., Chang C.J., Lin C.S., Lu C.C., Ko Y.F., Tseng S.F., Lai H.C., Young J.D. (2017). Anti-obesogenic and antidiabetic effects of plants and mushrooms. Nat. Rev. Endocrinol..

[2] Zhu R., Liu H., Liu C., Wang L., Ma R., Chen B., Li L., Niu J., Fu M., Zhang D. (2017). Cinnamaldehyde in diabetes: A review of pharmacology, pharmacokinetics and safety. Pharmacol. Res..

[3] Ghorbani Z., Hekmatdoost A., Mirmiran P. (2014). Anti-hyperglycemic and insulin sensitizer effects of turmeric and its principle constituent curcumin. Int. J. Endocrinol. Metab..

[4] Khan A., Safdar M., Ali Khan M.M., Khattak K.N., Anderson R.A. (2003). Cinnamon improves glucose and lipids of people with type 2 diabetes. Diabetes Care.

[5] Chuengsamarn S., Rattanamongkolgul S., Luechapudiporn R., Phisalaphong C., Jirawatnotai S. (2012). Curcumin extract for prevention of type 2 diabetes. Diabetes Care.

[6] Gonzalez E., McGraw T.E. (2009). The Akt kinases: Isoform specificity in metabolism and cancer. Cell Cycle.

[7] Urasaki Y., Beaumont C., Talbot J.N., Hill D.K., Le T.T. (2020). Akt3 Regulates the Tissue-Specific Response to Copaiba Essential Oil. Int. J. Mol. Sci..

[8] Chen W.S., Xu P.Z., Gottlob K., Chen M.L., Sokol K., Shiyanova T., Roninson I., Weng W., Suzuki R., Tobe K. (2001). Growth retardation and increased apoptosis in mice with homozygous disruption of the Akt1 gene. Genes. Dev..

[9] Cho H., Mu J., Kim J.K., Thorvaldsen J.L., Chu Q., Crenshaw E.B., Kaestner K.H., Bartolomei M.S., Shulman G.I., Birnbaum M.J. (2001). Insulin resistance and a diabetes mellitus-like syndrome in mice lacking the protein kinase Akt2 (PKB beta). Science.

[10] Tschopp O., Yang Z.Z., Brodbeck D., Dummler B.A., Hemmings-Mieszczak M., Watanabe T., Michaelis T., Frahm J., Hemmings B.A. (2005). Essential role of protein kinase B gamma (PKB gamma/Akt3) in postnatal brain development but not in glucose homeostasis. Development.

[11] George S., Rochford J.J., Wolfrum C., Gray S.L., Schinner S., Wilson J.C., Soos M.A., Murgatroyd P.R., Williams R.M., Acerini C.L. (2004). A family with severe insulin resistance and diabetes due to a mutation in AKT2. Science.

[12] Miao R., Fang X., Wei J., Wu H., Wang X., Tian J. (2022). Akt: A Potential Drug Target for Metabolic Syndrome. Front. Physiol.

[13] Iacovides D.C., Johnson A.B., Wang N., Boddapati S., Korkola J., Gray J.W. (2013). Identification and quantification of AKT isoforms and phosphoforms in breast cancer using a novel nanofluidic immunoassay. Mol. Cell Proteom..

[14] Guo H., Gao M., Lu Y., Liang J., Lorenzi P.L., Bai S., Hawke D.H., Li J., Dogruluk T., Scott K.L. (2014). Coordinate phosphorylation of multiple residues on single AKT1 and AKT2 molecules. Oncogene.

[15] Schrotter S., Leondaritis G., Eickholt B.J. (2016). Capillary Isoelectric Focusing of Akt Isoforms Identifies Highly Dynamic Phosphorylation in Neuronal Cells and Brain Tissue. J. Biol. Chem..

[16] Aspinall-O’Dea M., Pierce A., Pellicano F., Williamson A.J., Scott M.T., Walker M.J., Holyoake T.L., Whetton A.D. (2015). Antibody-based detection of protein phosphorylation status to track the efficacy of novel therapies using nanogram protein quantities from stem cells and cell lines. Nat. Protoc..

[17] Urasaki Y., Beaumont C., Workman M., Talbot J.N., Hill D.K., Le T.T. (2020). Potency Assessment of CBD Oils by Their Effects on Cell Signaling Pathways. Nutrients.

[18] Chen J.Q., Wakefield L.M., Goldstein D.J. (2015). Capillary nano-immunoassays: Advancing quantitative proteomics analysis, biomarker assessment, and molecular diagnostics. J. Transl. Med..

[19] Urasaki Y., Fiscus R.R., Le T.T. (2016). Molecular classification of fatty liver by high-throughput profiling of protein post-translational modifications. J. Pathol..

[20] Urasaki Y., Pizzorno G., Le T.T. (2016). Chronic Uridine Administration Induces Fatty Liver and Pre-Diabetic Conditions in Mice. PLoS ONE.

[21] Urasaki Y., Beaumont C., Workman M., Talbot J.N., Hill D.K., Le T.T. (2020). Fast-acting and receptor-mediated regulation of neuronal signaling pathways by copaiba essential oil. Int. J. Mol. Sci..

[22] Kostrzewa T., Przychodzen P., Gorska-Ponikowska M., Kuban-Jankowska A. (2019). Curcumin and Cinnamaldehyde as PTP1B Inhibitors With Antidiabetic and Anticancer Potential. Anticancer. Res..

[23] Han X., Xu B., Beevers C.S., Odaka Y., Chen L., Liu L., Luo Y., Zhou H., Chen W., Shen T. (2012). Curcumin inhibits protein phosphatases 2A and 5, leading to activation of mitogen-activated protein kinases and death in tumor cells. Carcinogenesis.

[24] Haystead T.A., Sim A.T., Carling D., Honnor R.C., Tsukitani Y., Cohen P., Hardie D.G. (1989). Effects of the tumour promoter okadaic acid on intracellular protein phosphorylation and metabolism. Nature.

[25] Wiesmann C., Barr K.J., Kung J., Zhu J., Erlanson D.A., Shen W., Fahr B.J., Zhong M., Taylor L., Randal M. (2004). Allosteric inhibition of protein tyrosine phosphatase 1B. Nat. Struct. Mol. Biol..

[26] Song G., Ouyang G., Bao S. (2005). The activation of Akt/PKB signaling pathway and cell survival. J. Cell. Mol. Med..

[27] Kumar C.C., Madison V. (2005). AKT crystal structure and AKT-specific inhibitors. Oncogene.

[28] Risso G., Blaustein M., Pozzi B., Mammi P., Srebrow A. (2015). Akt/PKB: One kinase, many modifications. Biochem. J..

[29] Sarbassov D.D., Guertin D.A., Ali S.M., Sabatini D.M. (2005). Phosphorylation and regulation of Akt/PKB by the rictor-mTOR complex. Science.

[30] Bellacosa A., Chan T.O., Ahmed N.N., Datta K., Malstrom S., Stokoe D., McCormick F., Feng J., Tsichlis P. (1998). Akt activation by growth factors is a multiple-step process: The role of the PH domain. Oncogene.

[31] Conus N.M., Hannan K.M., Cristiano B.E., Hemmings B.A., Pearson R.B. (2002). Direct identification of tyrosine 474 as a regulatory phosphorylation site for the Akt protein kinase. J. Biol. Chem..

[32] Chen R., Kim O., Yang J., Sato K., Eisenmann K.M., McCarthy J., Chen H., Qiu Y. (2001). Regulation of Akt/PKB activation by tyrosine phosphorylation. J. Biol. Chem..

[33] Facchinetti V., Ouyang W., Wei H., Soto N., Lazorchak A., Gould C., Lowry C., Newton A.C., Mao Y., Miao R.Q. (2008). The mammalian target of rapamycin complex 2 controls folding and stability of Akt and protein kinase C. EMBO J..

[34] Ding L., Zhang L., Biswas S., Schugar R.C., Brown J.M., Byzova T., Podrez E. (2017). Akt3 inhibits adipogenesis and protects from diet-induced obesity via WNK1/SGK1 signaling. JCI Insight.

[35] Santi S.A., Lee H. (2010). The Akt isoforms are present at distinct subcellular locations. Am. J. Physiol. Cell. Physiol..

[36] Yuan X., Han L., Fu P., Zeng H., Lv C., Chang W., Runyon R.S., Ishii M., Han L., Liu K. (2018). Cinnamaldehyde accelerates wound healing by promoting angiogenesis via up-regulation of PI3K and MAPK signaling pathways. Lab. Investig..

[37] Shakibaei M., Mobasheri A., Buhrmann C. (2011). Curcumin synergizes with resveratrol to stimulate the MAPK signaling pathway in human articular chondrocytes in vitro. Genes. Nutr..

[38] Huang B., Yuan H.D., Kim D.Y., Quan H.Y., Chung S.H. (2011). Cinnamaldehyde prevents adipocyte differentiation and adipogenesis via regulation of peroxisome proliferator-activated receptor-gamma (PPARgamma) and AMP-activated protein kinase (AMPK) pathways. J. Agric. Food Chem..

[39] Kim T., Davis J., Zhang A.J., He X., Mathews S.T. (2009). Curcumin activates AMPK and suppresses gluconeogenic gene expression in hepatoma cells. Biochem. Biophys. Res. Commun..

[40] Stevens N., Allred K. (2022). Antidiabetic Potential of Volatile Cinnamon Oil: A Review and Exploration of Mechanisms Using In Silico Molecular Docking Simulations. Molecules.

[41] Cocorocchio M., Baldwin A.J., Stewart B., Kim L., Harwood A.J., Thompson C.R.L., Andrews P.L.R., Williams R.S.B. (2018). Curcumin and derivatives function through protein phosphatase 2A and presenilin orthologues in Dictyostelium discoideum. Dis. Model. Mech..

[42] Johnson T.O., Ermolieff J., Jirousek M.R. (2002). Protein tyrosine phosphatase 1B inhibitors for diabetes. Nat. Rev. Drug Discov..

[43] Sangodkar J., Farrington C.C., McClinch K., Galsky M.D., Kastrinsky D.B., Narla G. (2016). All roads lead to PP2A: Exploiting the therapeutic potential of this phosphatase. FEBS J..

[44] Bononi A., Agnoletto C., De Marchi E., Marchi S., Patergnani S., Bonora M., Giorgi C., Missiroli S., Poletti F., Rimessi A. (2011). Protein kinases and phosphatases in the control of cell fate. Enzyme Res..

[45] Liao Y., Hung M.C. (2010). Physiological regulation of Akt activity and stability. Am. J. Transl. Res..

[46] Li G., Ji X.D., Gao H., Zhao J.S., Xu J.F., Sun Z.J., Deng Y.Z., Shi S., Feng Y.X., Zhu Y.Q. (2012). EphB3 suppresses non-small-cell lung cancer metastasis via a PP2A/RACK1/Akt signalling complex. Nat. Commun..

[47] Ugi S., Imamura T., Maegawa H., Egawa K., Yoshizaki T., Shi K., Obata T., Ebina Y., Kashiwagi A., Olefsky J.M. (2004). Protein phosphatase 2A negatively regulates insulin’s metabolic signaling pathway by inhibiting Akt (protein kinase B) activity in 3T3-L1 adipocytes. Mol. Cell. Biol..

[48] He X., Li M., Yu H., Liu G., Wang N., Yin C., Tu Q., Narla G., Tao Y., Cheng S. (2020). Loss of hepatic aldolase B activates Akt and promotes hepatocellular carcinogenesis by destabilizing the Aldob/Akt/PP2A protein complex. PLoS Biol..

[49] Ravichandran L.V., Chen H., Li Y., Quon M.J. (2001). Phosphorylation of PTP1B at Ser(50) by Akt impairs its ability to dephosphorylate the insulin receptor. Mol. Endocrinol..

[50] Shulman G.I. (2000). Cellular mechanisms of insulin resistance. J. Clin. Invest.

