# Supplementary material for: Cinnamaldehyde and Curcumin Prime Akt2 for Insulin-Stimulated Activation
Source: Nutrients. 2022 Aug 12;14(16):3301. doi: 10.3390/nu14163301 (PMC9416494; doi:10.3390/nu14163301)
Supplement: Supplementary file 1 [file nutrients-14-03301-s001.zip › nutrients-1852879-supplementary.pdf]

nutrients MDPI

Supplementary Materials

Cinnamaldehyde and Curcumin Prime Akt2 for Insulin-Stimulated Activation

Yasuyo Urasaki[1] & Thuc T Le[1,] *
[1]College of Pharmacy, Roseman University of Health Sciences, 10530 Discovery Drive, Las Vegas, NV 89135, U.S.A.
*Correspondence: Thuc T. Le, tle5@roseman.edu

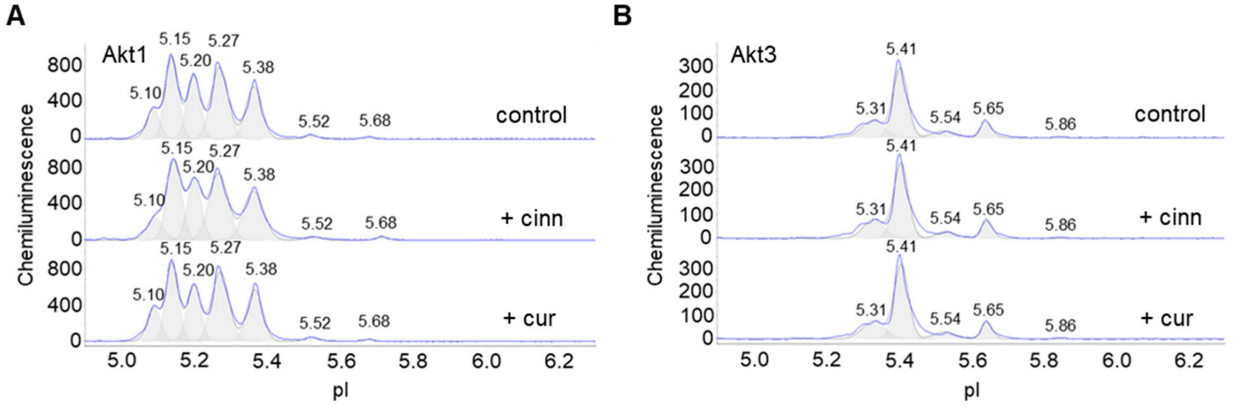

Figure S1. Cinnamaldehyde and curcumin have no observable effect on Akt1 and Akt3. (**A**) Distribution of Akt1 isoform as a function of isoelectric points in untreated control preadipocytes (top electropherogram), or preadipocytes treated with cinnamaldehyde (middle electropherogram) or curcumin (bottom electropherogram). (**B**) Distribution of Akt3 as a function of isoelectric points in untreated control preadipocytes (top electropherogram), or preadipocytes treated with cinnamaldehyde (middle electropherogram) or curcumin (bottom electropherogram).

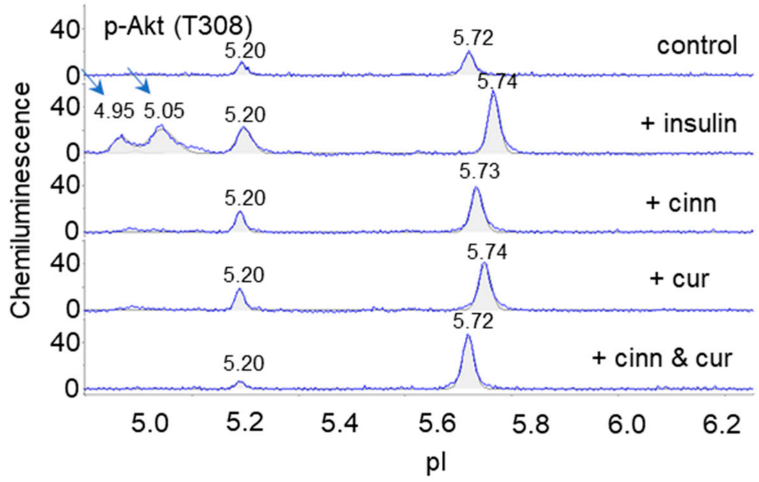

Figure S2. Insulin induces the appearance of p-Akt (T308). Distribution of p-Akt (T308) as a function of isoelectric points in untreated control preadipocytes (top electropherogram), or preadipocytes treated with insulin (second electropherogram), cinnamaldehyde (third electropherogram), curcumin (fourth electropherogram), or combined cinnamaldehyde and curcumin (bottom electropherogram). Arrows point to the appearance of new peaks following treatment versus untreated control.

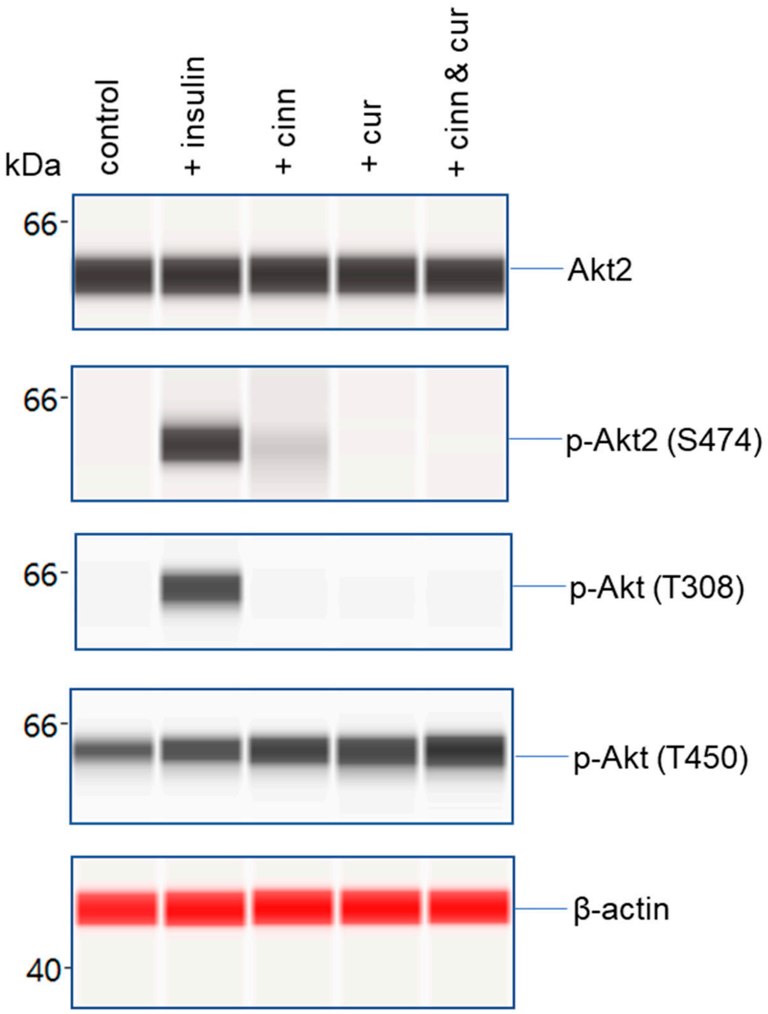

Figure S3. Cinnamaldehyde and curcumin increase p-Akt (T450) isoform. Capillary Western assays of Akt2 (top panel), p-Akt2 (S474) (second panel), p-Akt (T308) (third panel), p-Akt (T450), and β-actin (bottom panel) in untreated control preadipocytes (first lane), or in preadipocytes treated with insulin (second lane), cinnamaldehyde (third lane), curcumin (fourth lane), or combined cinnamaldehyde and curcumin (fifth lane). Akt2 and β-actin serve as loading controls.

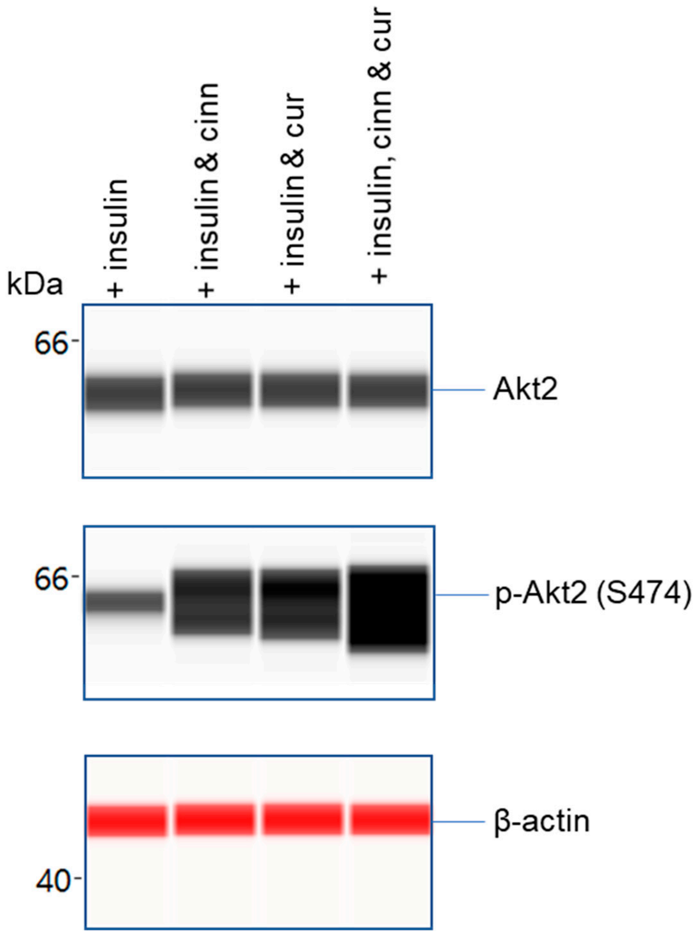

Figure S4. Cinnamaldehyde and curcumin enhance insulin-stimulated phosphorylation of Akt2 (S474). Capillary Western assays of Akt2 (top panel), p-Akt2 (S474) (middle panel), and β-actin (bottom panel) in preadipocytes treated with insulin only (first lane), insulin and cinnamaldehyde (second lane), insulin and curcumin (third lane), and combined insulin, cinnamaldehyde and curcumin (fourth lane). Akt2 and β-actin serve as loading controls.

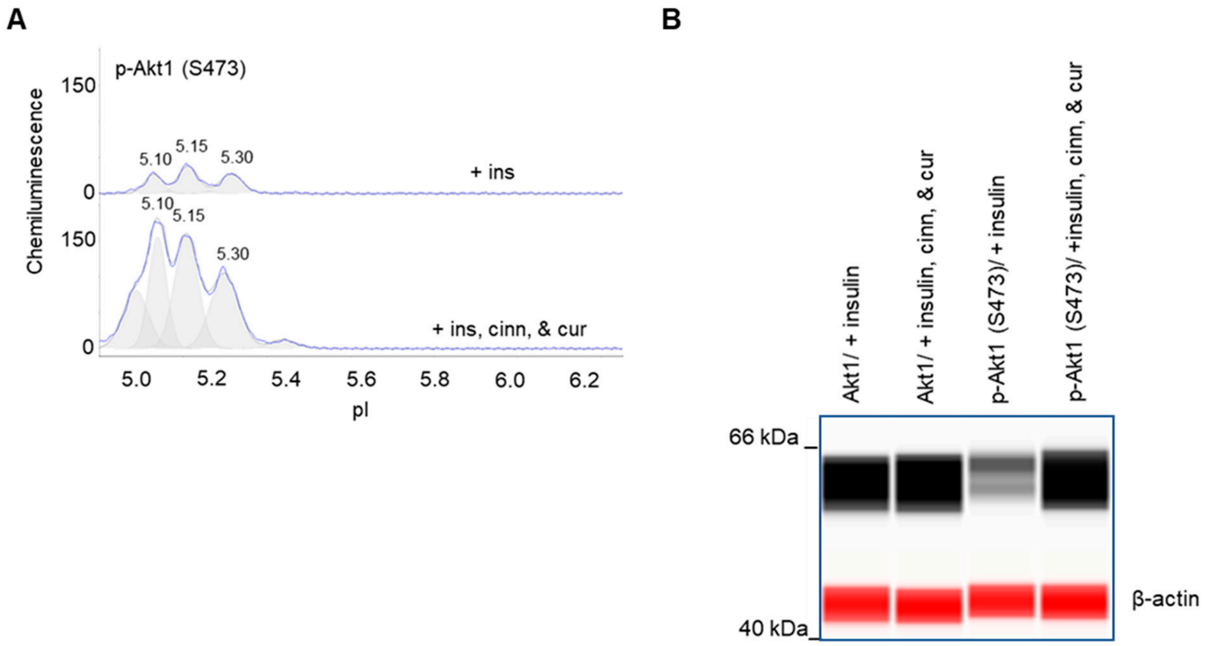

Figure S5. Cinnamaldehyde and curcumin enhance insulin-stimulated activation of Akt1. (**A**) Distribution of p-Akt1 (S473) as a function of pI values in preadipocytes treated with insulin alone (top electropherogram) or with insulin together with cinnamaldehyde and curcumin (bottom electropherogram). (**B**) Capillary Western immunoassays of: Akt1 in preadipocytes treated with insulin alone (first top lane) or with insulin together with cinnamaldehyde and curcumin (second top lane); p-Akt1 (S473) in preadipocytes treated with insulin alone (third top lane) or with insulin together with cinnamaldehyde and curcumin (fourth top lane). Akt1 (first and second top lanes) and β-actin (bottom row) serve as loading controls.

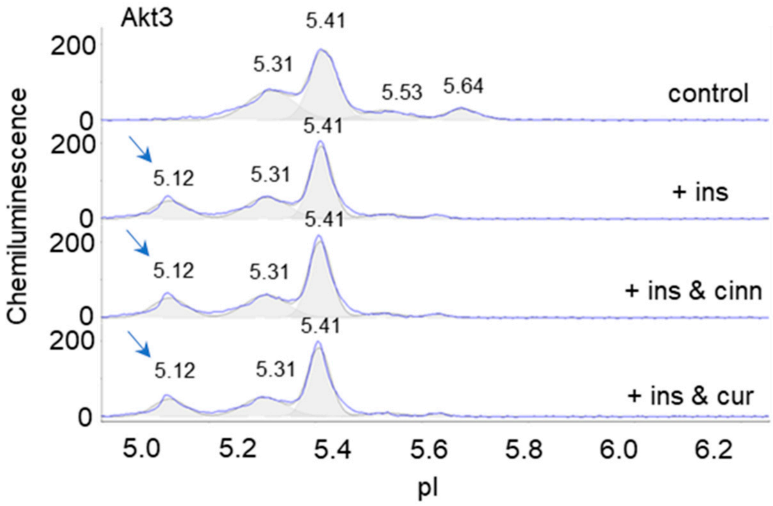

Figure S6. Cinnamaldehyde and curcumin have no effect on insulin-stimulated phosphorylation of Akt3. Distribution of Akt3 as a function of pI values in untreated control preadipocytes (top electropherogram) or in preadipocytes treated

with insulin (second electropherogram), insulin and cinnamaldehyde (third electropherogram), or insulin and curcumin (fourth electropherogram). Arrows point to an Akt3 phosphoisoform that appears following the treatment with insulin.

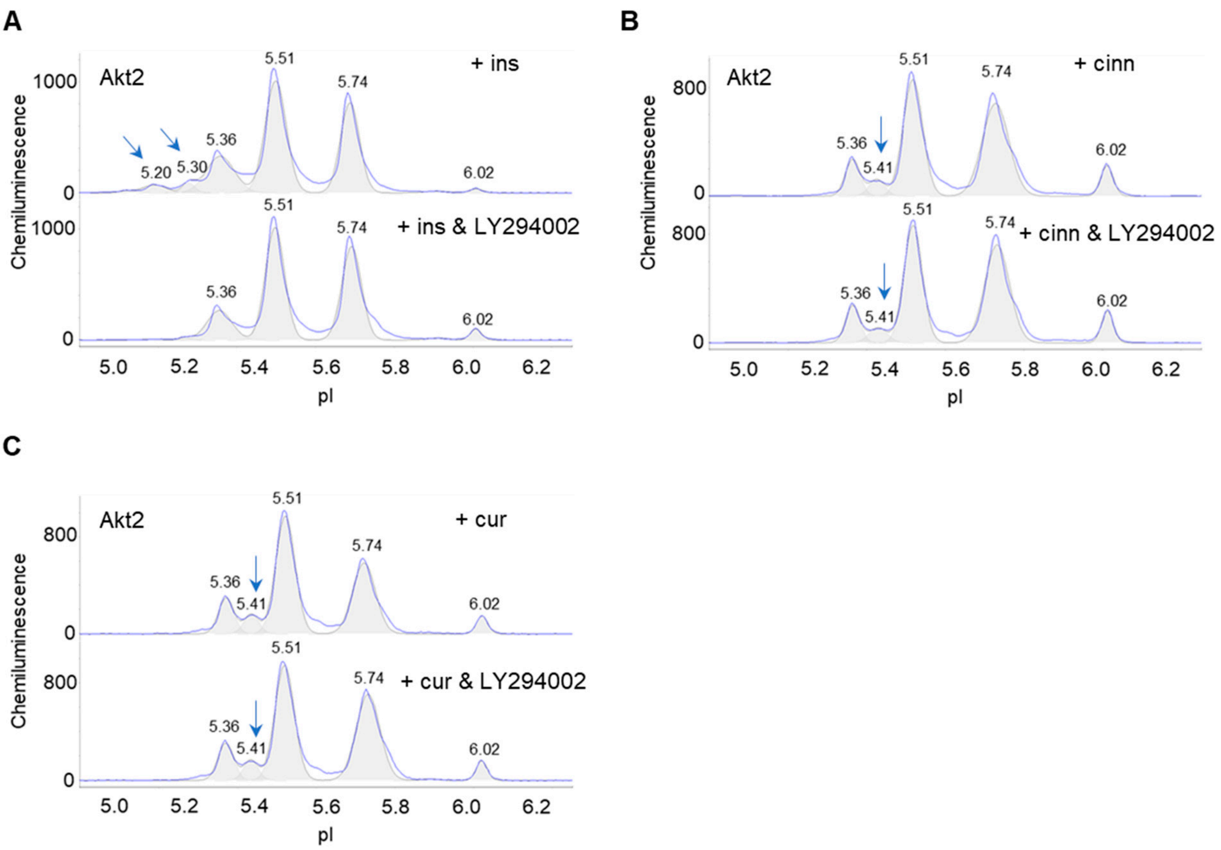

Figure S7. The effects of cinnamaldehyde and curcumin are independent of insulin signaling pathway. (**A**) Distribution of Akt2 as a function of pI values in preadipocytes treated with insulin (top electropherogram) or insulin and LY294002 (bottom electropherogram). (**B**) Distribution of Akt2 as a function of pI values in preadipocytes treated with cinnamaldehyde (top electropherogram) or cinnamaldehyde and LY294002 (bottom electropherogram). (C) Distribution of Akt2 as a function of pI values in preadipocytes treated with curcumin (top electropherogram) or curcumin and LY294002 (bottom electropherogram). Arrows point to Akt2 peaks that appear following treatment with insulin, cinnamaldehyde, or curcumin. LY294002, a PI3K inhibitor, was used at a final concentration of 50 µM.

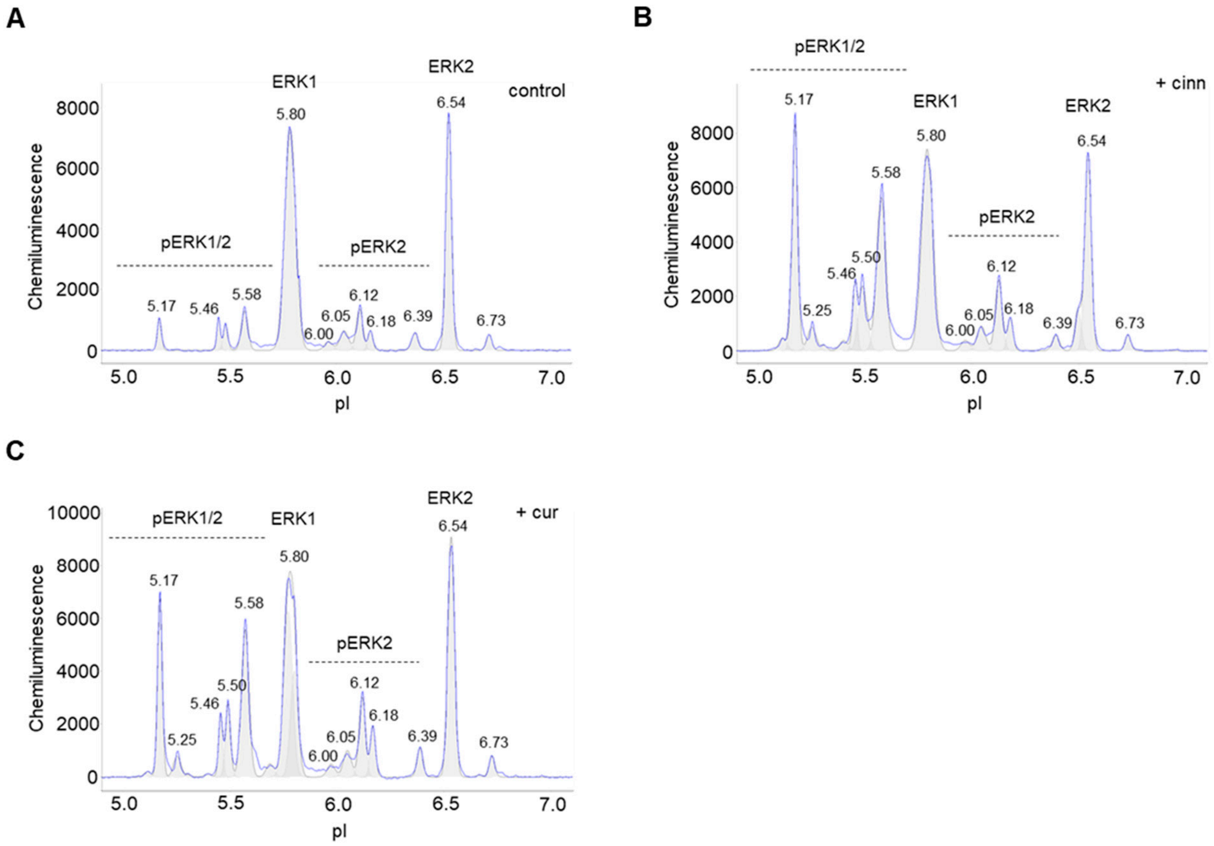

Figure S8. Cinnamaldehyde and curcumin promote ERK1/2 phosphorylation. (**A**) Distribution of ERK1/2 as a function of pI values in control untreated preadipocytes. (**B**) Distribution of ERK1/2 as a function of pI value in preadipocytes treated with cinnamaldehyde. (**C**) Distribution of ERK1/2 as a function of pI values in preadipocytes treated with cu
